# Galanin stimulates neurite outgrowth from sensory neurons by inhibition of Cdc42 and Rho GTPases and activation of cofilin

**DOI:** 10.1111/jnc.12379

**Published:** 2013-08-22

**Authors:** Sally-Ann Hobson, Penny A Vanderplank, Robert J P Pope, Niall C H Kerr, David Wynick

**Affiliations:** 1 Schools of Physiology and Pharmacology and Clinical Sciences, University of BristolBristol, UK

**Keywords:** Cdc42, cofilin, DRG, galanin, Rho

## Abstract

We and others have previously shown that the neuropeptide galanin modulates neurite outgrowth from adult sensory neurons via activation of the second galanin receptor; however, the intracellular signalling pathways that mediate this neuritogenic effect have yet to be elucidated. Here, we demonstrate that galanin decreases the activation state in adult sensory neurons and PC12 cells of Rho and Cdc42 GTPases, both known regulators of filopodial and growth cone motility. Consistent with this, activated levels of Rho and Cdc42 levels are increased in the dorsal root ganglion of adult galanin knockout animals compared with wildtype controls. Furthermore, galanin markedly increases the activation state of cofilin, a downstream effector of many of the small GTPases, in the cell bodies and growth cones of sensory neurons and in PC12 cells. We also demonstrate a reduction in the activation of cofilin, and alteration in growth cone motility, in cultured galanin knockout neurons compared with wildtype controls. These data provide the first evidence that galanin regulates the Rho family of GTPases and cofilin to stimulate growth cone dynamics and neurite outgrowth in sensory neurons. These findings have important therapeutic implications for the treatment of peripheral sensory neuropathies.

Damage to the sensory neurons of the dorsal root ganglion (DRG) induce major and long lasting changes in the expression of a large number of genes that promote neurite outgrowth and axonal regeneration (for review see Navarro *et al*. 2007). Under favourable conditions, for instance following a crush injury, most nerve fibres successfully regenerate. However, in many clinically relevant circumstances, reduced or disordered axonal regeneration often results in a loss of sensation and/or the development of chronic neuropathic pain states. The physiological mechanisms that underlie injury-induced axonal regeneration are therefore of considerable scientific and clinical importance.

The principal secreted factors that are involved in the regeneration of peripheral sensory neurons include the neurotrophins, members of the TGFβ superfamily, cytokines and a number of neuropeptides. We have previously demonstrated that the neuropeptide galanin plays a trophic role to the DRG and stimulates neurite outgrowth and regeneration of injured sensory neurons (Holmes *et al*. 2000; Mahoney *et al*. 2003). In the adult, galanin is expressed at low levels in < 5% of DRG neurons, which are predominantly small diameter C-fibres (Hokfelt *et al*. 1987). Following nerve damage, there is a very substantial increase (up to 120-fold) in the levels of galanin in the rodent, primate and human DRG (Hokfelt *et al*. 1987; Wiesenfeld *et al*. 1992) and the peptide is then abundantly expressed in ∼ 30–40% of sensory neurons (Hokfelt *et al*. 1994). Following a crush injury to the sciatic nerve regeneration is reduced by 35% in galanin knockout (Gal-KO) mice compared with wildtype (WT) controls (Holmes *et al*. 2000). This impaired regenerative capacity is paralleled by a similar reduction in neurite outgrowth of dispersed adult mouse DRG neurons (Holmes *et al*. 2000), which can be rescued by the addition of exogenous galanin (Mahoney *et al*. 2003). The neuritogenic role played by galanin is mediated via activation of galanin receptor 2 (GalR2) in a protein kinase C (PKC) dependant manner (Hobson *et al*. 2006).

When an axon is severed it produces a highly motile tip called a growth cone, which senses the surrounding environment and if favourable leads to axonal elongation. This leads to replacement of the distal nerve segment that is lost or damaged, promoting reinnervation of the target organ and thus recovery of function (Allodi *et al*. 2012). The most distal portion of the growth cone extends both lamellipodia, which are veil like membranous protrusions, and finger like extensions known as filopodia. These protrusions are mainly formed by actin filament bundles which are in equilibrium between actin polymerisation and depolymerisation generating the protrusion force necessary to allow the filopodia to explore the microenvironment. The small GTPases of the Rho subfamily have been shown to be key mediators of the interaction between cell adhesion molecules and the cytoskeleton and are now regarded as major regulators of axonal and dendritic growth (Luo 2000; Auer *et al*. 2011), integrating upstream signalling cues with downstream cytoskeletal rearrangements.

Rho GTPases are a subfamily of the Ras superfamily of small GTPases which were first found to mediate filopodia and lamellipodial formation in fibroblasts (Ridley *et al*. 1992; Nobes and Hall 1995). The Rho GTPases act as molecular switches and can exist in two states: an inactive GDP-bound state and an active GTP-bound state. In the active GTP-bound form, the activated GTPase binds its effectors and thus elicits various biological activities. Each of the three major members of the Rho subfamily, Rho, Rac and Cdc42 have been found to play a specific role in axonal and dendritic morphology. RhoA is involved in growth cone retraction in response to collapsing guidance cues (Jalink *et al*. 1994; Kozma *et al*. 1997; Thies and Davenport 2003), whilst Rac and Cdc42 promote neurite outgrowth by formation of lamellipodia and filopodia respectively (Ridley *et al*. 1992; Nobes and Hall 1995). Rac and Cdc42 share a common effector: p21-activated kinase (Pak) (Manser *et al*. 1994; Burbelo *et al*. 1995), which may explain some of the common phenotypes seen when the activities of Rac or Cdc42 are perturbed (Manser *et al*. 1994). Pak phosphorylates LIM kinase (LIMK) (Edwards *et al*. 1999), which in turn phosphorylates and inhibits the actin-binding protein cofilin. Cofilin can also be phosphorylated by the Rho pathway via Rho-associated coiled-coil-containing kinase (ROCK)1 and ROCK2 phosphorylation of LIMK2 (Sumi *et al*. 1999).

Cofilin is the principal terminal effector of a number of signalling cascades (that includes many of the superfamily of small GTPases), and its activation leads to cytoskeletal rearrangement. It is expressed at high levels in the growth cones of neurons, colocalises with filamentous (F) actin (Bamburg and Bray 1987) and over-expression leads to increased neurite outgrowth (Meberg and Bamburg 2000). Cofilin is an important regulator of actin dynamics and acts by increasing actin depolymerisation and severing filaments (Chen *et al*. 2000). Whether cofilin promotes the assembly or disassembly of actin depends on the concentration of cofilin relative to actin as well as the concentration of other binding proteins (Van *et al*. 2008). The major mechanism by which cofilin activity is regulated is via phosphorylation at Serine 3 (Ser3) which inhibits its binding to actin monomers and thus reduces its actin depolymerising activity (Morgan *et al*. 1993; Agnew *et al*. 1995).

In this study, we have characterised an intracellular signalling pathway by which galanin stimulates neurite outgrowth from sensory neurons which has important therapeutic implications for the treatment of peripheral sensory neuropathies. We show here that galanin reduces the activation state of both Rho and Cdc42 GTPases, but does not affect the activation state of Rac. The reduction in the GTP-bound form of these small GTPases leads to a marked increase in the activation of the actin-binding protein cofilin with associated changes in growth cone dynamics which in turn promotes neuritogenesis.

## Materials and methods

### Animals

All experiments were performed on 8-week-old female homozygous Gal-KO mice and strain, age and sex-matched 129OlaHsd WT controls. Details of the strain and breeding history have been previously described (Wynick *et al*. 1998; Mahoney *et al*. 2003). In brief, Gal-KO mice were generated using the E14 cell line. A PGK-*Neo* cassette in reverse orientation was used to replace exons 1–5, and the mutation was bred to homozygosity and has remained inbred on the 129olaHsd strain. All animals were fed standard chow and water *ad libitum* and maintained on a 12 h on 12 h off light schedule. Animal care procedures were performed within University and UK Home Office protocols and guidelines and compliant with the ARRIVE guidelines.

### PC12 cell culture

PC12 cells (a kind gift from Dr S Allen, University of Bristol, UK) were cultured on collagen-(Sigma, Gillingham, UK) coated culture flasks in high glucose Dulbecco's modified Eagle's medium (Invitrogen, Paisley, UK) supplemented with 10% horse serum (Sigma), 5% fetal bovine serum (Sigma), penicillin (10 000 units/mL) and streptomycin (10 mg/mL) (Sigma) and 2 mM l-glutamine (Invitrogen) at 37°C in a humidified incubator with 95% air 5% CO_2_. Cells were differentiated in Dulbecco's modified Eagle's media as above with 1% fetal bovine serum and 1 ng/mL nerve growth factor (Sigma) for 48 h.

### Protein extraction and western blotting

Dissected DRG were homogenised using a micropestle in a 1.5 mL Eppendorf in cold extraction buffer, 25 mM Tris.HCl pH 7.2, 150 mM NaCl, 5 mM MgCl_2_, 1% NP-40 and 5% glycerol supplemented with phosphatase and protease inhibitor cocktails (Sigma). PC12 cells were removed from the culture flasks using a cell scraper into cold extraction buffer. Cells were incubated on ice for 5 min and centrifuged at 16 000 *g* at 4°C for 15 min. Equal amounts of protein were determined using BCA protein assay kit (Thermo Scientific, Loughborough, UK) and separated by sodium dodecyl sulfate–polyacrylamide gel electrophoresis (SDS-PAGE) and transferred to Hybond C Extra (GE Healthcare, Little Chalfont, UK) nitrocellulose membrane. Proteins were detected using the following antibodies anti-cofilin (1 : 5000) (Ma *et al*. 2009) and anti-phospho(Ser3)-cofilin (1 : 1000) antibody (Gu *et al*. 2010) (Abcam, Cambridge, UK) and anti-α-tubulin (Schwamborn *et al*. 2007) (1 : 2000) (Sigma) followed by the appropriate secondary antibody anti-mouse peroxidase (1 : 1000) (Vector Laboratories, Peterborough, UK), anti-rabbit peroxidise (1 : 2000) (Cell Signalling technologies, Boston, MA, USA). Blots were developed using SuperSignal West Pico Chemiluminescent substrate (Thermo Scientific). α-tubulin was used as a loading control. Semiquantitative analysis was performed using Scion Image software (Scion Corp, Frederick, MD, USA). Values were expressed as percentage of control samples normalised to tubulin loadings. In each case, results were obtained from three independent experiments.

### GTPase activation assays

PC12 cells were treated with 1 μM galanin (Bachem, St Helens, UK) for various times (0, 5 or 10 min) as indicated and then assayed for GTPase activity. For active GTPase levels in WT and Gal-KO animals, DRG were dissected from eight animals and homogenised in ice cold extraction buffer as above. Activated RhoA, Rac1 and Cdc42 (Hu *et al*. 2012) were isolated by immunoprecipitating the cell lysates for 1 h with either the Rho-binding domain (RBD) of Rhotekin fused with GST (GST-Rhotekin-RBD) or the p21-binding domain (PBD) of p21-activated kinase 1 (GST-Pak1-PBD) for both Cdc42 and Rac (Thermo Scientific). Proteins were separated by SDS-PAGE as above and probed with either an anti-Rho (1 : 600), anti-Rac1 (1 : 1000) or anti-Cdc42 (1 : 250) antibody (Thermo Scientific) followed by the appropriate horseradish peroxidase-conjugated secondary antibody and developed using SuperSignal West Femto Chemiluminescent substrate (Thermo Scientific). Total lysates were also separated by SDS-PAGE. Semiquantitative analysis was performed using Scion Image software. Values were expressed as percentage of control samples.

### DRG culture

Cultures were performed as described previously (Holmes *et al*. 2000). Adult female 8-week-old mice were killed by cervical dislocation and DRG from the lumbar, thoracic and cervical regions were removed aseptically, trimmed of connective tissue and nerve roots, and pooled in F12 medium (Sigma). Ganglia were subjected to 0.25% collagenase P (Roche, Burgess Hill, UK) for 1 h at 37°C, washed in phosphate-buffered saline (PBS) and treated enzymatically with trypsin-EDTA (Sigma) for 10 min at 37°C. Ganglia were washed in medium containing trypsin inhibitor and then mechanically dissociated by trituration using a flame-narrowed Pasteur pipette. After centrifugation cells were resuspended in F12 media supplemented with 5% horse serum, 1 mM glutamine, and 10 ng/mL gentamicin. To enhance the cultures for neurons and eliminate much of the cellular debris cells were plated on six well plates coated with 0.5 mg/mL polyornithine and maintained overnight at 37°C in a humidified incubator with 95% air- 5% CO_2_ (Patrone *et al*. 1999). Medium was then removed and discarded. Neurons were removed from the surface by squirting with a jet of fresh medium. Following centrifugation cells were plated onto appropriate sized culture plates treated with 0.5 mg/mL polyornithine and 5 μg/mL laminin and maintained at 37°C in a humidified incubator with 95% air, 5% CO_2_.

### Growth cone analysis

For measurement of growth cone size, DRG neurons were fixed using 4% paraformaldehyde for 20 min at 20°C. Cells were washed twice with PBS and then permeabilised with 0.1% Triton X-100 in PBS for 5 min. Cells were washed twice with PBS and then stained with alexafluor 488 phalloidin (Invitrogen) for 30 min at 20°C. Cells were washed three times with PBS and mounted with Vectashield (Vector laboratories). Images were acquired using a Leica DM4000B microscope using a 63x oil immersion objective and a LeicaDC500 (Leica, Milton Keynes, UK) camera. Scion Image was used to measure the area of the distal 12 μm portion of the growth cone. At least 225 growth cones were analysed from three independent experiments.

### Ratio imaging of total and phospho(Ser3)cofilin

DRG neurons were fixed using 4% paraformaldehyde for 20 min at 20°C, washed twice with PBS and permeabilised with 0.1% Triton X-100 in PBS for 5 min. Following blocking for 1 h in normal donkey serum (Sigma) cells were incubated with anti-phospho(Ser3) cofilin (1 : 100) (Hu *et al*. 2012; Wang *et al*. 2012) antibody (Abcam) overnight at 4°C. Cells were washed three times with PBS and then incubated with anti-rabbit Cy3 (1 : 400) (Jackson Laboratories, West Grove, PA, USA) for 1 h at 20°C. Following three washes with PBS, a Vector M.O.M. fluorescein immunodetection kit (Vector Laboratories) was used according to the manufacturers protocol using a monoclonal anti-total cofilin (1 : 200) antibody (Wang *et al*. 2012) (Abcam). Coverslips were mounted using Vectashield (Vector Laboratories). Images were acquired using a Leica SP5 confocal microscope using an oil immersion 63x objective. Image analysis was performed using Volocity software (Improvision, Coventry, UK). The distal 12 μm of growth cones was selected and the ratio of the intensity of the phospho(Ser3) image over the total cofilin intensity for individual growth cones was calculated. At least 160 growth cones were analysed from three independent experiments.

### Timelapse video microscopy

DRG neurons were plated onto 35 mm glass bottomed microwell dish (MatTek, Ashland, MA, USA). Phase contrast images were collected using a Leica DMIRB inverted microscope fitted with a Hamamatsu CCD camera using a 40x oil immersion objective. Cells were maintained at 37°C in Leibovitz's L15 media (Sigma). Images were taken every 20 s during a 30 min recording period. Filopodial dynamics were quantified by measuring extension or retraction of individual filopodia during sequential frames using Volocity software (Improvision). At least 72 filopodia were analysed from three independent experiments.

### Statistical analysis

Data are presented as the mean ± SEM. Unless otherwise stated unpaired Student's *t*-test was used to analyse statistical significance. *p* < 0.05 was considered significant. To analyse differences between genotypes at different time points in timelapse filopodial extension/retraction studies a two way anova was performed with Bonferroni *post hoc* test. For western blots with more than 2 two groups, a one way anova was performed with a Dunnett's *post hoc* test.

## Results

### Galanin modulates the activation of Cdc42 and Rho

We have previously shown that galanin increases neurite outgrowth in adult murine sensory neurons via activation of the G_q_ coupled receptor, GalR2 (Holmes *et al*. 2000; Mahoney *et al*. 2003; Hobson *et al*. 2006). As there is a growing body of evidence that G_q_ coupled receptors can activate the Rho family of GTPases (Chikumi *et al*. 2002; Dutt *et al*. 2002; Usui *et al*. 2003; Vogt *et al*. 2003; Notcovich *et al*. 2010), we therefore wished to study whether galanin regulates the activation states of the major members of the Rho family, namely Rho, Rac and Cdc42. Rho family GTPases cycle between an active GTP-bound state and an inactive GDP-bound state. Activity can therefore be determined by a pull-down assay where the amount of the GTPase that is bound to the specific binding domain of a downstream effector is quantified (Ren *et al*. 1999). As pull-down assays require significant amounts of cell lysate, we initially used PC12 cells, a rat adrenal medulla pheochromocytoma cell line (Greene and Tischler 1976), to optimise Rho, Rac and Cdc42 pull-down assays. PC12 cells are commonly used as a model of neurite outgrowth and galanin has been shown to stimulate neurite outgrowth in this cell line (Hawes *et al*. 2006).

We confirmed the presence of endogenous GalR2 receptors in PC12 cells. Real-time quantitative RT-PCR of undifferentiated PC12 cells detected the expression of moderate levels of GalR2 mRNA (25.50 cycles), whereas GalR3 and galanin transcripts were rare (35.79 and 38.43 cycles respectively) and GalR1 mRNA was not detected by 40 cycles. The expression levels of the four transcripts did not significantly change 48 h after nerve growth factor-induced differentiation of PC12 cells (see Supplemental data S1).

**Figure 1:**
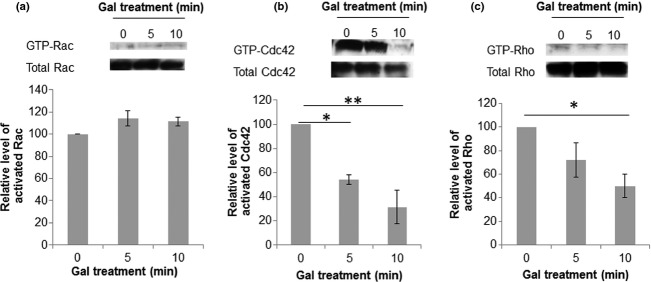
Treatment with galanin reduces levels of activated Cdc42 and Rho, but not Rac in PC12 cells. Representative images of western blots and corresponding densitometric quantification of the level of activated (a) Rac, (b) Cdc42 and (c) Rho in PC12 cells. Results were obtained from three independent experiments, *p < 0.05, **p < 0.01, anova with Dunett's *post hoc* test, error bars represent SEM.

We performed pull-down assays for each of the major Rho family of small GTPases and tested whether their activation state altered following 5 and 10 min treatment with galanin. Treatment of PC12 cells with 1 μM galanin appeared to have no effect on the activation state of Rac (Fig. 1a). In contrast, treatment with galanin for 10 min produced a 69 ± 14% reduction in GTP-Cdc42 levels (Fig. 1b) and a 50 ± 9.7% decrease in the amount of GTP-Rho (Fig. 1c).

**Figure 2:**
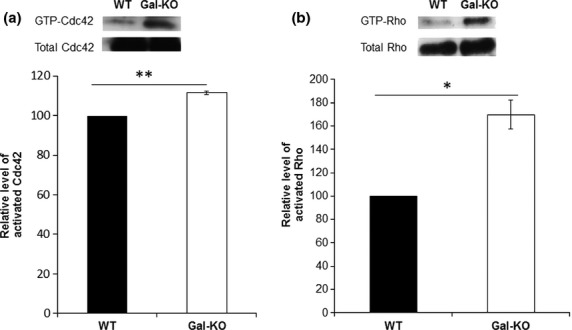
Levels of activated Cdc42 and Rho in adult mouse dorsal root ganglion (DRG). Representative image of western blots and corresponding densitometric quantification of the level of activated (a) Cdc42 and (b) Rho in adult mouse DRG. Galanin knockout (Gal-KO) animals demonstrate higher levels of both activated Cdc42 and Rho than WT controls. Results were obtained from three independent experiments, *p < 0.05, **p < 0.01, *t*-test, error bars represent SEM.

Having optimised the pull-down assays, we then tested whether the loss of galanin in the adult Gal-KO DRG alters the levels of the GTP-bound forms of these Rho proteins. Results demonstrate that Gal-KO mice have significantly higher levels of both GTP-Cdc42 (Fig. 2a) and GTP-Rho (Fig. 2b) than WT controls, consistent with our findings in PC12 cells.

### Galanin regulates the activation state of cofilin

**Figure 3:**
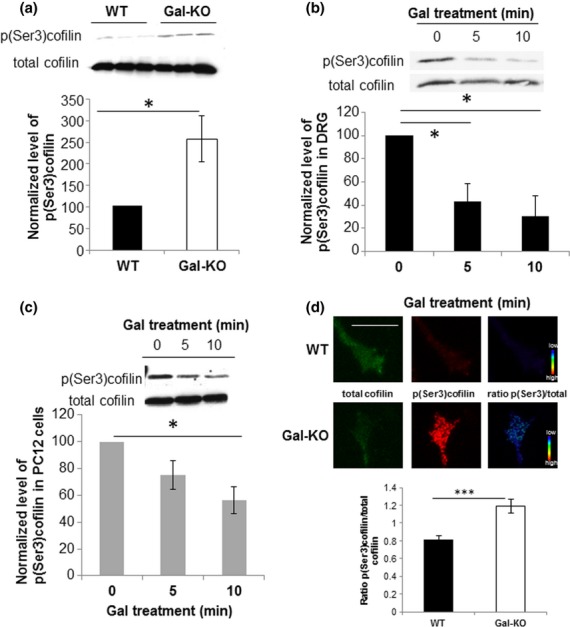
Levels of expression of p(Ser3)cofilin in dorsal root ganglion (DRG) and PC12 cells. (a) Representative images of western blots and corresponding densitometric quantification of the level of expression of p(Ser3)cofilin in WT and Galanin knockout (Gal-KO) DRG. Gal-KO DRG expressed significantly increased levels of p(Ser3)cofilin compared with WT controls. Protein levels were normalised to tubulin controls. Results were obtained from three independent experiments, *p < 0.05, Student's *t*-test, error bars represent SEM. (b) Representative images of western blots and corresponding densitometric quantification showing the reduction in the level of expression of p(Ser3)cofilin in WT DRG following galanin treatment. Results were obtained from three independent experiments, *p < 0.05, anova with Dunnett's *post hoc* test, error bars represent SEM. (c) The effect of galanin on p(Ser3)cofilin levels in PC12 cells mirror its effects in the DRG. Representative images of western blots and corresponding densitometric quantification showing the reduction in the level of expression of p(Ser3)cofilin in PC12 cells following galanin treatment. Results were obtained from three independent experiments, *p < 0.05, anova with Dunnett's *post hoc* test, error bars represent SEM. (d) Quantification of the average p(Ser3)cofilin/total cofilin ratio in the distal 12 μm of the growth cone of WT and Gal-KO DRG neurons. Measurements were taken from at least 160 growth cones obtained from three independent experiments, ***p < 0.001, *t*-test, error bars represent SEM. Representative images of WT and Gal-KO growth cones are shown stained for total cofilin, p(Ser3)cofilin along with the intensity ratio of p(Ser3)cofilin/total cofilin shown in pseudocolour scale. Scale bar, 10 μm.

Many factors that promote neurite extension have been shown to alter the activation state of the actin-binding protein cofilin (Meberg *et al*. 1998). Phosphorylation of cofilin is regulated by LIMK which is under the control of the Rho family of small GTPases (reviewed in Bamburg 1999). We therefore studied the levels of phospho(Ser3)cofilin relative to total cofilin in DRG from Gal-KO mice and WT controls by western blotting. Levels of the inactive phospho(Ser3)cofilin were 2.5 ± 0.53-fold higher in Gal-KO animals than in WT controls (Fig. 3a), indicating a reduction in cofilin activity in the Gal-KO DRG.

To confirm that the difference in phospho(Ser3)cofilin levels seen in the Gal-KO was because of the lack of galanin in the adult animal, we treated cultured adult WT sensory neurons and differentiated PC12 cells with 1 μM galanin for 5 and 10 min. Results demonstrate a rapid and significant decrease (44 ± 10%)in the levels of phospho(Ser3) cofilin relative to total cofilin over a 10-min period in the DRG (Fig. 3b) and PC12 cells (Fig. 3c), consistent with an increase in cofilin activation.

We next asked whether galanin affects the activation state of cofilin within individual growth cones (as opposed to the levels in the cell bodies of the sensory neurons, as the process of extirpation removes the soma from the axons). We performed quantitative immunofluorescence and measured the ratio of phospho(Ser3)cofilin to total cofilin in the distal 12 μm in the DRG growth cones. Our findings demonstrate that Gal-KO growth cones contain 46 ± 9% more phospho(Ser3)cofilin to total cofilin than in WT growth cones (Fig. 3d). The finding that both the growth cones and the cell bodies of Gal-KO DRG have an increase in inactive phospho(Ser3)cofilin is again consistent with the previously reported reduction in neurite outgrowth in cultured Gal-KO sensory neurons.

### Galanin modulates filopodial dynamics and growth cone area

**Figure 4:**
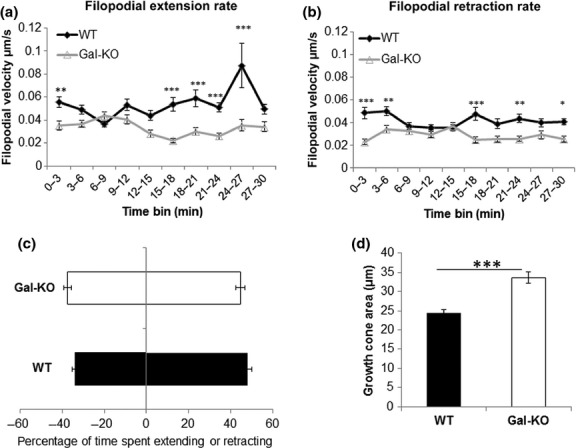
Growth cone dynamics in WT and Galanin knockout (Gal-KO) adult mouse dorsal root ganglion (DRG) neurons. Timelapse video microscopy was used to analyse the filopodial extension (a) and retraction (b) rates. Measurements were taken from at least 72 filopodia obtained from three independent experiments, *p < 0.05, **p < 0.01, ***p < 0.001, two way anova followed by Bonferroni *post hoc* analysis, error bars represent SEM. (c) The percentage of time that filopodia on the growth cone spent extending and retracting was analysed taking data from at least 72 filopodia obtained from three independent experiments, error bars represent SEM. (d) Growth cone area of the distal 12 μm of neurite was measured, demonstrating that Gal-KO have significantly larger growth cones than WT controls. Measurements were taken from at least 225 growth cones obtained from three independent experiments, ***p < 0.001, *t*-test, error bars represent SEM.

As galanin alters the activation states in the DRG of Cdc42, Rho and cofilin which are known to influence filopodial extension, growth cone retraction and cytoskeletal rearrangement, we then studied whether the absence of galanin also affected filopodial dynamics. We used timelapse video microscopy to study the extension and retraction rates of filopodia from cultured DRG neurons over a period of 30 min. Both the extension and retraction rates of filopodia were significantly reduced in the Gal-KO cultures compared with WT cultures (Fig. 4a and b). We also analysed the percentage of time that each filopodia spent extending or retracting, but found no significant differences between the genotypes (Fig. 4c).

We then tested whether the absence of galanin affects growth cone area, by measuring the area of the distal 12 μm of the growth cones of WT and Gal-KO DRG neurons. Results demonstrate a 37 ± 6% increase in growth cone area in Gal-KO neurons compared with WT controls (Fig. 4d).

## Discussion

We have previously shown that the impaired regenerative capacity observed in Gal-KO mice is paralleled by a 35% reduction in neurite length, compared with WT controls (Holmes *et al*. 2000). The deficits in neurite outgrowth can be rescued by the addition of either exogenous galanin or the GalR2/3 specific agonist Gal2-11 (Mahoney *et al*. 2003). Our finding that galanin plays a neuritogenic role is supported by work in rat dispersed DRG cultures. Galanin significantly increased both axonal elongation and also the number of branch points (Suarez *et al*. 2006). Furthermore, treatment of foetal DRG cultures with galanin increased growth cone velocity by nearly twofold compared with controls (Sanford *et al*. 2008). To confirm which galanin receptor subtypes mediate the effects of galanin, we studied GalR2-KO animals, demonstrating a marked reduction in neurite outgrowth identical to that observed in the Gal-KO animals (Hobson *et al*. 2006). The addition of galanin (which also activates GalR1 and GalR3) failed to rescue the deficits in the GalR2-KO cultures (Hobson *et al*. 2006) implying that neither GalR1 nor GalR3 play a major role in galanin-mediated neuritogenesis of adult sensory neurons. All three galanin receptor subtypes couple to G_i/o_ leading to an inhibition of adenylate cyclase (Habert-Ortoli *et al*. 1994; Smith *et al*. 1998). GalR2 additionally signals via G_q/11_ to activate phospholipase C and protein kinase C (PKC) (Habert-Ortoli *et al*. 1994; Wang *et al*. 1998; Wittau *et al*. 2000). Consistent with galanin modulation of neurite outgrowth via GalR2 in DRG neurons, the neuropeptide has also been shown to stimulate neurite outgrowth in a PKC dependant manner from PC12 cells and neurospheres derived from adult hippocampal progenitor cells (Mahoney *et al*. 2003; Hawes *et al*. 2006; Hobson *et al*. 2006). The intracellular signalling pathways which mediate the galanin/GalR2-dependent increase in neurite outgrowth in sensory neurons have yet to be elucidated and are the subject of this study.

Here, we demonstrate that galanin decreases activated levels of Rho (Fig. 1c) and Gal-KO animals have significantly higher levels of GTP-Rho than WT controls (Fig. 2b). Rho has been implicated in regulating growth cone collapse and its activation leads to neurite retraction (Jalink *et al*. 1994; Kozma *et al*. 1997; Leclere *et al*. 1997; Lehmann *et al*. 1999). This effect is thought to be mediated via ROCK1 and ROCK2 phosphorylation of LIMK2 which phosphorylates cofilin, inhibiting its activity (Agnew *et al*. 1995; Arber *et al*. 1998; Maekawa *et al*. 1999; Gehler *et al*. 2004b). Thus, the finding that Gal-KO animals have higher levels of active Rho than their WT controls may explain in part the deficits seen in regeneration *in vivo* and neurite outgrowth *in vitro* (Holmes *et al*. 2000).

Our finding that galanin also regulates activated levels of Cdc42 (Figs 1b and 2a) is in accordance with published work demonstrating that Cdc42 regulates filopodial formation in response to extracellular cues in non-neuronal cells (Nobes and Hall 1995) and neuroblastoma cells (Kozma *et al*. 1997). The currently accepted Cdc42 signalling pathway in neurons is via activation of Pak and LIMK which increase the levels of inactive phospho(Ser3)cofilin (Agnew *et al*. 1995; Arber *et al*. 1998; Edwards *et al*. 1999; Maekawa *et al*. 1999; Govek *et al*. 2005). Here, we show that galanin decreases the activated levels of Cdc42 (Fig. 1b) and that Gal-KO animals have significantly higher levels of GTP-Cdc42 than WT controls (Fig. 2a). Consistent with these findings, two recent studies using cultured cortical and hippocampal neurons have demonstrated that inhibition of Cdc42 signalling is critical to dendritic branching and neurite outgrowth via activation of cofilin (Peris *et al*. 2012; Rosario *et al*. 2012). Of note these findings are contrary to previous published data showing over-expression of constitutively active Cdc42 (CAcdc42) constructs increases filopodial length and number in retinal ganglion cells (Chen *et al*. 2006) and the number of filopodia and increased rate of neurite outgrowth in chick spinal cord neurons (Brown *et al*. 2000). However, some studies have demonstrated that Cdc42 (like other GTPases) may need to complete a full GTP-binding/GTP hydrolysis cycle to propagate signals (Luo 2000; Fidyk *et al*. 2006), whereas CaCdc42 is GTP hydrolysis defective. Results using dominant negative constructs (DNCdc42) have been less clear with some studies showing DNCdc42 decreased neurite outgrowth (Threadgill *et al*. 1997) whilst others showed no significant effect on neurite outgrowth (Brown *et al*. 2000). DNGTPase constructs act by sequestering multiple guanine nucleotide exchange factors (GEFs), but most of these GEFs are not specific for a single RhoGTPase, and thus the DN mutant affects other RhoGTPase pathways (Czuchra *et al*. 2005; Pertz *et al*. 2008). Furthermore, knockdown of Cdc42 in neuroblastoma cells by siRNA rather than DNconstruct actually led to a significant increase in neurite length (Pertz *et al*. 2008). Thus, whilst CA and DN constructs have been important in the analysis of RhoGTPase functions in cells, data should be interpreted with caution and it is important to analyse the endogenous RhoGTPase activation states.

Irrespective of the pathways by which Rac, Rho and Cdc42 GTPases modulate cofilin activation, there is good agreement in the literature that it plays a key role in the control of actin dynamics and neurite outgrowth (Meberg and Bamburg 2000). The cofilin family is ubiquitously expressed in eukaryotes and the activity of all vertebrate cofilin proteins is regulated by phosphorylation. Phosphorylation at Ser3 inhibits the binding of cofilin to actin monomers and its actin depolymerising activity (Morgan *et al*. 1993; Agnew *et al*. 1995). Expression of cofilin is abundant within neuronal growth cones (Bamburg and Bray 1987) and signals which alter growth cone motility have been shown to alter phosphorylation of cofilin (Bamburg and Bray 1987; Meberg *et al*. 1998). Actin monomer is abundantly available in DRG growth cones and polymerisation occurs rapidly when free F-actin barbed ends become available for monomer addition by cofilin severing. Thus, activated (unphosphorylated) cofilin positively correlates with neurite outgrowth (Meberg *et al*. 1998; Kuhn *et al*. 2000; Meberg and Bamburg 2000). We show here that following treatment with galanin the levels of phospho(Ser3)cofilin fall in DRG and PC12 cells indicating that galanin activates cofilin (Fig. 3b and c). These data are also in keeping with the finding that treatment with other factors which are trophic to neurons such as nerve growth factor and brain-derived neurotrophic factor (BDNF) also decrease cofilin phosphorylation and promote neurite outgrowth (Gehler *et al*. 2004b; Chen *et al*. 2006; Endo *et al*. 2007).

Since galanin signals through the Rho, Cdc42 and cofilin pathways, we then asked whether Gal-KO animals have altered growth cone dynamics compared to WT controls. Gal-KO sensory growth cones extended and retracted their filopodia at a significantly reduced rate compared with WT filopodia (Fig. 4a and b), consistent with a previous study which showed an increased velocity of rat DRG growth cones in the presence of galanin (Sanford *et al*. 2008). The neurotrophin BDNF has also been shown to increase filopodial extension and retraction rates by reducing activation of RhoA (Gehler *et al*. 2004a) and activating cofilin (Gehler *et al*. 2004b). Most recently, knockout of RhoE (a member of the atypical family of Rho proteins) has been shown to increase RhoA and Cdc42 activation in hippocampal neurons, associated with an decrease in cofilin activation and a reduced rate of filopodial extension and retraction (Peris *et al*. 2012). In contrast to these data, expression of CACdc42, albeit in drosophila, increased extension and retraction rates by 52% (Kim *et al*. 2002) and Cdc42-KO mice display an inhibition in the amount of filopodia/min formed and retracted (Garvalov *et al*. 2007). Both these studies are in embryonic animals and the apparent discordance in findings could be as a result of differing roles played by Cdc42 in early as compared with the later stages of development, as suggested by Rosario *et al*. (2012).

The increase in activation of Rho, Cdc42 and inactive p(Ser3)cofilin in Gal-KO mice and the resulting reduction in the rates of filopodial extension and retraction may explain in part, the observed increase in growth cone area compared with WT controls (Fig. 4a). Of note, growth cone area was also larger in Cdc42-KO embryonic hippocampal neurons than WT controls (Garvalov *et al*. 2007). Whilst this may seem contradictory with our data at the level of Cdc42, it is consistent with the unexpected increased levels of phospho-LIMK and p(Ser3)cofilin observed in these mice (Garvalov *et al*. 2007).

In summary, we have characterised an intracellular pathway by which galanin stimulates neurite outgrowth in sensory neurons, demonstrating a role for the neuropeptide in growth cone dynamics and morphology via modulation of the activation states of Rho and Cdc42 GTPases and the actin-binding protein cofilin. These findings have important therapeutic implications for the treatment of peripheral sensory neuropathies.

## Abbreviations

CA: constitutively active
DN: dominant negative
DRG: dorsal root ganglion
F: filamentous
Gal-KO: galanin knockout
GalR2: Galanin receptor 2
GEF: guanine nucleotide exchange factor
GST: glutathione S-transferase
LIMK: LIM kinase
Pak: p21-activated kinase
PKC: protein kinase C
RBD: Rho binding domain
ROCK: Rho-associated coiled-coil containing kinase
Ser: Serine
TGFβ: transforming growth factor β
WT: wildtype

## Supporting information

Data S1 Real time quantitative RT-PCR assays.
