# Supplementary material for: Galanin stimulates neurite outgrowth from sensory neurons by inhibition of Cdc42 and Rho GTPases and activation of cofilin
Source: J Neurochem. 2013 Aug 22;127(2):199–208. doi: 10.1111/jnc.12379 (PMC3935412; doi:10.1111/jnc.12379)
Supplement: Data S1 — Real time quantitative RT-PCR assays. [file jnc0127-0199-Sd1.docx]

**Supplemental Data**

*Real-time quantitative RT-PCR assays* – Relative mRNA expression levels were derived by the comparative threshold cycle (C_t_) method, as previously described (1). RNA was purified from PC12 cells either undifferentiated or differentiated with 1 ng/ml NGF for 2 days (each *n* = 3; RNeasy Mini Kit, Qiagen), then treated with RQ1 DNase (Promega), and 2 μg total RNA was incubated with reverse transcriptase (RT+) or without enzyme (RT-, control for RT-dependence) in 100 μl (Taqman Reverse Transcription Reagents, Applied Biosystems), from which triplicate RT-PCRs were set up.

The endogenous control glyceraldehyde 3-phosphate dehydrogenase (Gapdh) primer and probe set sequences: forward primer 5'-CTTGTGACAAAGTGGACATTG-3', reverse primer 5'-CTGGAACATGTAGACCATGTAGTTGA-3' and non-extendable Taqman probe 5'-CCATCAACGACCCCTTCATTGAC-3' correspond, respectively, to nucleotides (nt) 140-160, 213-188 and 164-186 of the rat reference RNA (NM_017008). GalR1 primers and probe sequences: forward primer 5'-GTCAAAAAAGTCAGAGGCATCCAA-3', reverse primer 5'-GCCATGATATGCCAAATACCACAA-3' and Taqman probe 5'-AAAAGACTGCACAGACTGTCCTGGTGGTC-3' correspond, respectively, to nt 924-947, 1003-980 and 950-978 of the rat reference RNA (NM_012958). GalR2 primers and probe sequences: forward primer 5'-TCTGCAAGGCTGTTCATTTC-3', reverse primer 5'-TAGCGGATGGCCAGATACCT-3' and Taqman probe 5'-TTTCTCACTATGCACGCCAGCAGC-3' correspond, respectively, to nt 310-329, 406-387 and 336-359 of the rat reference RNA (NM_019172). GalR3 primers and probe sequences: forward primer 5'-GCTGGCGGCTCTCTTT-3', reverse primer 5'-TAGCCCGCGGCGAAGGT-3' and Taqman probe 5'-CCGTAGCGCACCGTGCCGT-3' correspond, respectively, to nt 469-484, 603-587 and 525-507 of the rat reference RNA (NM_019173). Galanin primers and probe sequences: forward primer 5'-CTGCTAGCCTGGCTCCTGTT-3', reverse primer 5'-TCCAGCCTCTCTTCTCCTTTGTT-3' and Taqman probe 5'-CCAGAGTGGCTGACAGGGTTGCAA-3' correspond, respectively, to nt 166-185, 247-225 and 211-188 of the rat reference RNA (NM_033237). The probes each had a 5' fluorescent reporter dye FAM (6-carboxy-fluorescein), except for the Gapdh probe with VIC, and all had the 3' quencher dye TAMRA (6-carboxy-tetramethyl-rhodamine) (Applied Biosystems).

All threshold cycle (C_t_) values were determined at the 0.2 default threshold, and values for undifferentiated PC12 cells are presented as mean ± S.E. In comparing undifferentiated to differentiated PC12 cells, relative expression levels were determined by the comparative C_t_ method (2) to endogenous control Gapdh levels. Results are presented as mean of log transformed data, with statistical significance judged by one-tail Student t test.

**Supplementary References**

1. Kerr, N. C., Gao, Z., Holmes, F. E., Hobson, S. A., Hancox, J. C., Wynick, D., and James, A. F. (2007) *Mol. Cell Neurosci.* **35,** 283-291

2. Giulietti, A., Overbergh, L., Valckx, D., Decallonne, B., Bouillon, R., and Mathieu, C. (2001) *methods* **25,** 386-401

qRT-PCR Cycle threshold (Ct) values for undifferentiated PC12 cells

GalR1 mRNA = not detected at 40 cycles

GalR2 mRNA = 25.499 (± 0.065; *n*=3) cycles

GalR3 mRNA = 35.788 (± 0.147; *n*=3) cycles

Galanin mRNA = 38.429 (± 0.246; *n*=3) cycles

Lack of effect of 48 hour differentiation of PC12 cells on specific transcripts

GalR1 mRNA = not detected at 40 cycles

GalR2 mRNA = no change (1.0938; *P* = 0.3994)

GalR3 mRNA = no change (0.8384; *P* = 0.3190)

Galanin mRNA = no change (0.8296; *P* = 0.4791)
